# Response to the comment on “Development of PancRISK, a urine biomarker-based risk score for stratified screening of pancreatic cancer patients”

**DOI:** 10.1038/s41416-020-1014-4

**Published:** 2020-08-03

**Authors:** Oleg Blyuss, Tatjana Crnogorac-Jurcevic

**Affiliations:** 1 School of Physics, Astronomy and Mathematics, University of Hertfordshire, Hatfield, UK; 2 Department of Paediatrics and Paediatric Infectious Diseases, Sechenov First Moscow University, Moscow, Russia; 3 Department of Applied Mathematics, Lobachevsky State University of Nizhny Novgorod, Nizhny Novgorod, Russia; 4 Centre for Molecular Oncology, Barts Cancer Institute, Queen Mary University of London, London, UK

**Keywords:** Tumour biomarkers, Cancer screening

We thank De Marco et al. for their valuable comments regarding TFF1, one of the biomarkers in our panel for early detection of pancreatic adenocarcinoma (PDAC).

We concur with the authors that TFF1 plays an important role in this malignancy, and find the information on the importance of copper binding to TFF1 of great interest. We have previously reported on deregulation of several proteins that bind copper in PDAC,^1,2^ and increased levels of this metal element have previously been described in both tissue and serum of patients with pancreatic malignancy.^3,4^ In fact, tissue copper levels were also found to be negatively associated with patient’s survival.^4^ This is not particularly surprising, considering the important role of this metal in cancer, as increased intra-tumoural copper and/or altered systemic copper distribution was shown to play an important role in cancer growth, angiogenesis, motility and metastases. In that respect, targeting copper in cancer therapy^5^ and testing metal complexes based on copper as a novel therapeutic approach (of note, platinum-based chemotherapy has been in use for over 40 years!), as highlighted by the authors, appears to be very promising.

Furthermore, we have recently reported that metal disbalance in PDAC can be detected via deregulated levels of several metals in urine, including increased level of urinary copper, which may potentially serve as a diagnostic and/or prognostic marker in pancreatic cancer.^6^ Thus, mechanistic studies on the effects of copper and its binding partners, as well as testing the potential of copper as novel therapeutic and a potential biomarker seem highly warranted.

## Data Availability

Not applicable.
